# The NIH Comparative Genomics Resource: addressing the promises and challenges of comparative genomics on human health

**DOI:** 10.1186/s12864-023-09643-4

**Published:** 2023-09-27

**Authors:** Kristin Bornstein, Gary Gryan, E. Sally Chang, Aron Marchler-Bauer, Valerie A. Schneider

**Affiliations:** 1 The MITRE Corporation, 7525 Colshire Dr, McLean, VA USA; 2 National Center for Biotechnology Information, National Library of Medicine, National Institutes of Health, Bethesda, MD 20894 USA

**Keywords:** Bioinformatics, Annotation, Human health, Zoonotic disease, Microbiome, Xenotransplantation, Oncology, Toxicology, NIH Comparative Genomics Resource (CGR), Sequence contamination

## Abstract

Comparative genomics is the comparison of genetic information within and across organisms to understand the evolution, structure, and function of genes, proteins, and non-coding regions (Sivashankari and Shanmughavel, Bioinformation 1:376-8, 2007). Advances in sequencing technology and assembly algorithms have resulted in the ability to sequence large genomes and provided a wealth of data that are being used in comparative genomic analyses. Comparative analysis can be leveraged to systematically explore and evaluate the biological relationships and evolution between species, aid in understanding the structure and function of genes, and gain a better understanding of disease and potential drug targets. As our knowledge of genetics expands, comparative genomics can help identify emerging model organisms among a broader span of the tree of life, positively impacting human health. This impact includes, but is not limited to, zoonotic disease research, therapeutics development, microbiome research, xenotransplantation, oncology, and toxicology. Despite advancements in comparative genomics, new challenges have arisen around the quantity, quality assurance, annotation, and interoperability of genomic data and metadata. New tools and approaches are required to meet these challenges and fulfill the needs of researchers. This paper focuses on how the National Institutes of Health (NIH) Comparative Genomics Resource (CGR) can address both the opportunities for comparative genomics to further impact human health and confront an increasingly complex set of challenges facing researchers.

## Background

Humans are ecologically and evolutionarily connected to many species on the planet. Humans are in constant interaction and competition with many species through the microbiome, pathogens, symbiotes, plants and animals, both directly and indirectly via the carbon cycle, nitrogen cycle, and all the food chains that we depend upon for survival. All eukaryotes, which feature compartmentalization of functions within and across specialized cells, share a common ancestor, even those organisms that are distantly related to humans [1]. Each species is well-adapted to their niches; they are survivors of previous life on the planet and have adaptations and capabilities affording them advantages in survival: hibernation, infectious disease tolerance, immune response, cancer survival, longevity, wound healing and limb regeneration, flight, bioelectricity, drought tolerance, sensory systems, and others. Comparing genomes is essential to understand these adaptations and how they contributed to evolutionary success. As more data becomes available and technology advances to permit more thorough analysis, comparative genomic findings in distantly related species, in addition to closely related ones, can be extrapolated to impact human health.

**Fig. 1:**
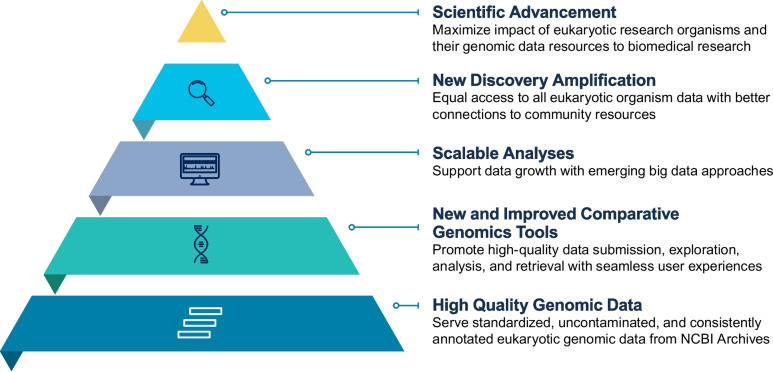
Benefits to the scientific community supported by Comparative Genomics Resource (CGR)

Comparative genomics is a rapidly developing field where the comparison of genetic information across and within species provides novel insights into many areas of biological investigation. However, there are a variety of data-related and technical challenges facing researchers, limiting the full realization of its potential [2]. The National Institutes of Health (NIH) Comparative Genomics Resource (CGR) aims to support comparative genomics by addressing these challenges and increasing the impact of this field, particularly on biomedical research (see Fig. 1). CGR facilitates reliable comparative genomics analyses for all eukaryotic organisms through community collaboration and a National Center for Biotechnology Information (NCBI) genomics toolkit. The toolkit provides high-quality data, tools, and interfaces for connecting community-provided resources with NCBI. CGR’s vision is to maximize the biomedical impact of eukaryotic research organisms and their genomic data resources to meet emerging research needs for human health [3].

**Fig. 2:**
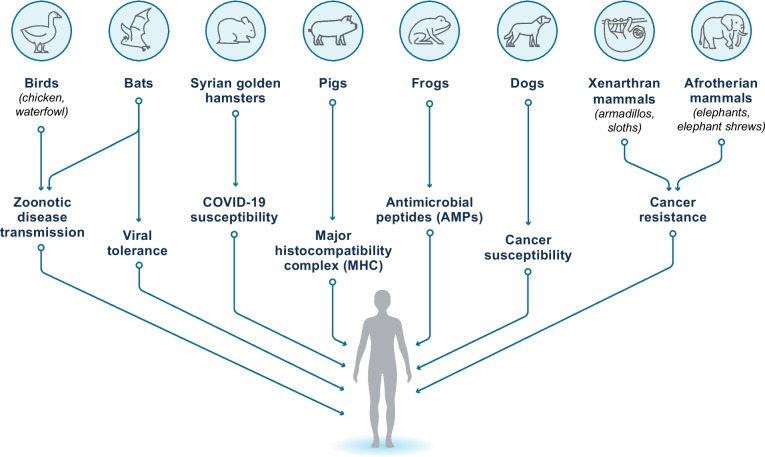
Examples of biomedically-relevant characteristics of organisms identified through comparative genomics research

This review provides examples of significant biological phenomena informed by comparative genomics that impact human health (see Fig. 2), presents challenges to this rapidly developing field, and indicates how CGR can meet those challenges. The expanding connection to sequenced organisms is integral to researching many of the traits of interest to human health (e.g., vision, metabolism) that may not be well-modeled in the most studied research organisms. Understanding the path not taken by the human species but by other species can elucidate evolved solutions to challenges those species confronted. Beyond the applications described in this paper, CGR is poised to support the comparative genomics field to capture and investigate biodiversity in ways that will have long-lasting repercussions including and beyond biomedical advances.

## Comparative genomics applications for human health

### Zoonotic diseases

Zoonotic infections are the spread of infectious disease from other species to humans and are a source of emerging infectious diseases (EIDs). Recent diseases of zoonotic origin such as avian influenza, COVID-19, Ebola, Zika, and HIV pose a significant public health threat; COVID-19 has killed 6.8 million worldwide as of April 2023 [4]. The likelihood of new EIDs is increasing due to several factors including greater international travel, which enables EIDs to reach populations around the globe, the expansion of humans into animal habitats, and the effects of deforestation and climate change forcing wild animals and humans to move into closer proximity. Even migratory birds play a role in global virus transmission [5–7].

Comparative genomics can provide the tools for studying the movement of infectious diseases across species and help investigate how pathogens adapt to hosts and barriers to “spillover” events where pathogens acquire mutations to infect other species [8]. In the case of SARS-CoV-2, there have been several outbreaks among animal species that raised concerns over disease reservoirs that could serve as points of potential spillover in the future. Naturally occurring infections or cases of human-to-animal transmission of SARS-CoV-2 have been documented in several domestic and wild animal species including cats, dogs, lions, tigers, mink, ferrets, snow leopards, pumas, and gorillas [9]. In the case of mink, documented spillover events describe where the virus transmitted back to humans and resulted in human-to-human transmission [10]. Similarly, influenza is endemic in a wide range of species (e.g., wild waterfowl, domestic poultry, swine, horses, dogs, bats, humans). The human health concerns involve not only spillover events directly from these reservoirs, but also cross-over events including an intermediate host, as was seen with H1N1, which was transmitted from birds to pigs to humans [11].

Additionally, comparative genomics can help identify gene differences that contribute to disease resistance and susceptibility across species and the key pathways involved in the immune response. The innate immune system and the adaptive immune system both play a role in the host’s response to infection, and studying immune responses in the transmission chain to humans is essential due to these differences in host immune responses [12]. For example, comparative genomics helped identify a range of mammals that could potentially be infected by SARS-CoV-2 via their angiotensin converting enzyme-2 (ACE2) proteins and serve as a route of animal-to-human transmission [13]. Specifically, Syrian Golden Hamsters were identified as having similar ACE2 proteins to humans and have since been used as a model organism for researching cytokine and chemokine profiles, antibody and adaptive immunity studies, and treatment responses [14].

The bat is another key organism of which various species have been linked to several zoonotic diseases. The bat immune system can harbor viruses and co-exist with them, an adaptation believed to allow bats to survive infection during hibernation when an immune response could cause a large caloric expenditure resulting in starvation [15, 16]. Bats are particularly important for discovering new viral threats; studying the bat virome is crucial to identifying both known viruses and novel viral threats. Because of their diet, bats can also harbor viruses for insects and plants as well as mammals; those non-mammalian viruses can impact agriculture and the food supply [17].

Agricultural intensification and environmental change have also been linked to emerging zoonotic diseases, as agricultural species encounter wildlife and can act as intermediaries between wildlife and humans [18]. Comparative genomics can help elucidate the role of agricultural species, such as pigs and chickens, in the transmission of diseases to humans. The comparative study of zoonotic diseases in these animals presents an opportunity to engineer animals that are resistant to zoonotic infections or create prophylactic vaccines for agricultural species as a forward line of defense.

Comparative genomics can support the battle against zoonotic disease by providing data and tools to discover potential EIDs before they jump to humans, aiding in the development of new diagnostics and identifying genes critical to host–pathogen interaction, which can inform the development of vaccines and countermeasures. Understanding the transmission of zoonotic disease through agricultural species can also help guide the development of preventative measures against those threats.

### Novel antimicrobial therapeutics

The World Health Organization (WHO) declared antimicrobial resistance as one of the top ten global public health threats [19]. Microbes are becoming resistant to existing drugs due to overuse and inappropriate use. In 2022, the WHO also reported that, since 2017, only 12 antibiotics have been approved, 10 of which belong to existing classes with established mechanisms of resistance [20]. The barriers to developing new antibiotics are high cost, low success rate, and a lengthy pathway to approval. In addition, resistance to new antibiotics is likely to appear, on average, within two to three years of market introduction [20]. The resultant shortage of effective antibiotics represents a threat to public health and the prospect of increased deaths due to infections that had been preventable in the recent past.

One way in which comparative genomics can contribute to the discovery of antimicrobial therapeutics is by helping discover novel antimicrobial peptides (AMPs) in newly sequenced organisms. More than 3,000 AMPs have been discovered, many of which are derived from eukaryotic organisms and have been cataloged in the Antimicrobial Peptide Database (APD) [21]. Other databases of AMPs include: Collection of Antimicrobial Peptides Release 4 (CAMPR4)—synthetic peptides [22], A Database of Anti-Microbial peptides (ADAM)—associating sequences with structures [23], Database of Antimicrobial Activity and Structure of Peptides version 3 (DBAASP)—structure and activity information [24], Data Repository of AntiMicrobial Peptides (DRAMP 3.0)—information on stapled AMPs which have modifications that brace them into stable conformations [25], and Linking AntiMicrobial Peptides 2 (LAMP2)—comprehensive information on AMPs and links to other AMP resources [26]. Not all AMPs are suitable for use as drugs for humans; stability (half-life), toxicity, and pharmacokinetics are all important factors, also known as ADME (absorption, distribution, metabolism, and excretion).

Frogs are currently the most studied model organisms for AMPs, with 30 percent of the peptides in the APD having been first identified in frogs. Each frog species can have a unique repertoire of peptides (usually 10–20) that differs even from closely-related species [27]. So far, no two species of frogs have the same assortment of peptides, and no identical sequences of peptides across species have been found, so the divergence across species is quite remarkable. AMPs often differ in physicochemical characteristics and mechanisms of action (MOA), making it difficult for microorganisms to develop resistance to this multidrug defense system. From a comparative genomics perspective, this provides a huge library of molecules to study the effect of structural changes on potency and may be useful in structure–activity relationship (SAR) studies for therapeutic development. AMPs have also been discovered in scorpions; some of those appear to have anti-viral activity [28–30]. Frog AMPs have been classified into 40 peptide families, with some additional “orphan AMPs” that do not fit into the 40 families [27]. The pre-pro region of the protein precursor of the AMPs shows remarkable conservation, while the C-terminal is highly divergent.

Comparative genomics can be leveraged in the development of AMPs as therapeutics by aiding in the identification of novel AMPs in newly sequenced organisms, facilitating the identification of protein families and evolutionary relationships of AMPs, and elucidating the relationship of structures and activity of AMPs. This includes searching newly sequenced organisms using motifs derived from conserved regions of known AMPs, looking at differential gene expression in tissues known to secrete AMPs, syntenic comparisons across related species in chromosomal regions where AMPs have previously been found, or developing new methods such as building models from CGR data using deep learning for finding candidate AMPs [31–34]. Machine learning is also being used for design and generation of novel synthetic AMPs [35]. The evolution of resistance to AMPs suggests that they likely evolve rapidly relative to other genes in the genome, which can aid in their discovery by focusing on evolutionary hot spots in the genome.

### Microbiomes

**Fig. 3:**
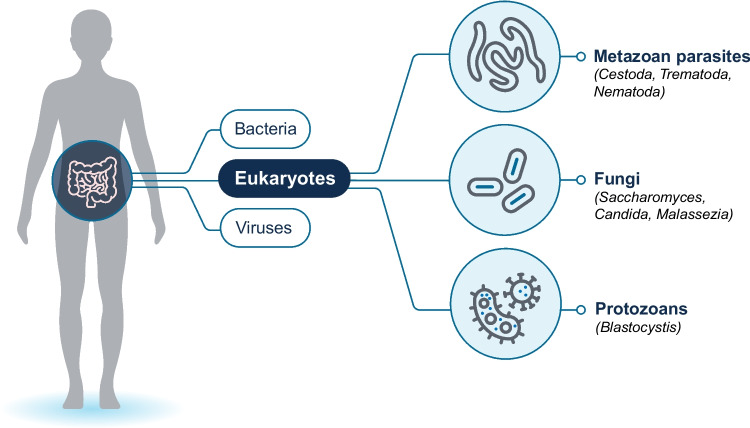
Microbial diversity in the human microbiome

The microbiome is a complex community of microorganisms that live together in a particular environment (see Fig. 3). The human microbiome is generally defined as those bacteria, fungi, parasites, and viruses that reside within various environments throughout the human body, from the skin to the intestines. Similar communities of microorganisms can be found across a diverse range of species, and comparative genomics can be leveraged to identify key differences in the microbiome of these species that may be responsible for host adaptation, immune mediation, metabolic function, and other complex issues impacting human health [36].

Much of the research regarding the microbiome heretofore has focused primarily on prokaryotic organisms. However, that belies the importance of eukaryotes such as metazoan parasites (e.g., cestodes, trematodes, nematodes), fungi (e.g., filamentous fungi and yeasts), and protozoans, within these complex microbial communities [37]. A fungal microbiome (“mycobiome”) was detected in more than 98 percent of fecal samples collected as part of the Human Microbiome Project, including *Saccharomyces, Candida,* and *Malassezia,* and the composition of the mycobiome differed by the social and geographic setting of the donor [38]. This underscores the complexity of the entire microbiome environment and the wide range of both external and internal factors that can impact its composition.

The relationship between *Pseudomonas aeruginosa* and *Candida albicans*, for instance, has well-documented fungal-bacterial cellular and metabolic interactions with far reaching human health impacts, such as cystic fibrosis in the lungs [39]. Meanwhile, just as bacterial components of the microbiome are highly impacted by antibiotic use and dietary exposures, the mycobiome has been affected both in the balance with affected bacterial populations and by exposure to antifungal agents. This has led to yeasts such as *C. albicans* to surge and elicit strong autoimmune responses in their human hosts [37]. Given the complex tapestry of external and internal factors affecting the microbiome, it is likely that antimicrobial resistance in prokaryotic members of the microbiome also has significant repercussions in eukaryotes. Such impacts have been documented in ocean microbial communities [40], and comparative genomics can be used to compare these marine findings to other microbiome communities.

Comparative genomics has also been used to explore the evolutionary relatedness of a wide range of fungal species and to examine the protein-encoding gene sequences to identify orthologs and paralogs among conserved regions. This helped to illuminate potential differences in gene innovations, gene family expansions, protein family diversification, and conservation of essential gene functions. For example, an unexpectedly high level of diversity was identified among genes involved in lipid metabolism. Furthermore, this line of investigation can help future research to apply transcriptome, proteome, and metabolome features from the well-characterized *S. cerevisiae* to other less well-characterized fungi [41]. Despite the well-characterized *S. cerevisiae* genome, it was historically difficult to determine if its detection in fecal samples was due to live colonization within microbiome or merely dead cells from dietary consumption. Comparative genomics was used to identify a homolog between *S. cerevisiae* to *C. albicans* (Yps7, an aspartyl protease) that is important for fungal growth on mucin of the human intestinal track, thus suggesting the viability of *S. cerevisiae* colonization in the human microbiome [42].

Outside of the mycobiome, comparative genomics studies combining metagenomics of protozoan species (such as *Blastocystis spp.*) with lifestyle metadata have profiled the role protozoan species play in increasing bacterial diversity [43] and impacting microbiome community composition [44]. Different *Blastocystis* species have been identified as beneficial or pathogenic within the human gut, and comparative genomics has been used to explore these different genomic and functional characteristics among *Blastocystis* subtypes. The research identified a strong association between the presence of any type of *Blastocystis* and the abundance and diversity of other microorganisms within the microbiome. Geographic origin of the sample and lifestyle of the donor were also associated with *Blastocystis* subtypes. Finally, *Blastocystis* colonization was found to be independent, if not negatively associated, with several morbidities including Crohn's disease and colorectal cancer [44].

Comparative genomics evaluating different subtypes, as described with *Blastocystis*, and within different species, such as different yeasts, has been integral in identifying differences between genetic and functional profiles that had not been possible in studies examining individual subtypes. The challenge of furthering knowledge of the eukaryotic impact in the microbiome is that these organisms are under-characterized, and the relationship between the community of microorganisms is extremely complex, dynamic, and affected by many factors.

### Xenotransplantation

Approximately 105,000 people are currently waiting for organ donations, roughly 80 percent of which are kidney donations [45]. There are two major problems with xenotransplantation: organ rejection by the host immune system and transfer of virus from the donor organism to the host. Several medical centers are collaborating with private sector companies to genetically engineer pigs that can donate organs to humans without rejection or retroviral transfer [46]; this technology is in an experimental phase in the clinic [47].

Fundamental similarities and differences have been found between pig and human genomics that have helped advance transplantation research. For example, both species have a dense gene cluster called the Major Histocompatibility Complex (MHC), as described by Renard et al. In humans this is referred to as the Human Leucocyte Antigen Complex (HLA) and in pigs as the Swine Leucocyte Antigen Complex (SLA). These MHC genes code for cell surface antigens that help an individual’s immune system distinguish self from non-self. The likelihood of transplant rejection is reduced when these antigens are similar between donor and host. Sequencing of the SLA found that within the 151 loci annotated, 28 genes (including all the SLA class I genes) had no unambiguous orthologs in humans; these are likely to be important in the divergence between pigs and humans. Comparative analysis with humans revealed the absence of HLA-A and other class I-like loci, the absence of HLA-DP like loci, and the separation of the extended and classical class II regions from the rest of the MHC by insertion of the centromere. The insertion of the centromere occurred within a cluster of butyrophilin-like (BTNL) genes located at the boundary of the class I and class II regions, which might have resulted in the loss of an ortholog to the human C6orf10 gene [48]. Mapping and sequencing of the MHC loci using bacterial artificial chromosomes (BACs) and their annotation also led to the mapping of Porcine Endogenous Retroviruses (PERVs), as discussed below [49, 50].

As described for the MHC, comparative genomics clarified how 80 million years of evolution diverged human and pig genomes, and how conserved and divergent elements contribute to the immune rejection response. Comparative genomics exposed the intricacies of the genome, complexity of the immune response where many genes are interacting in ways that are not obvious, and potential impacts of environmental factors, even when the exact function of the immune-related genes within the immune system remains unknown. The genetic background of the host was found to be vitally important in transplant success. The technical challenge is to achieve immune tolerance, which is the ability to transplant without using immunosuppressive drugs.

There are two approaches to introducing the genetic modifications to donor pigs: microinjection of CRISPR/CAS9 into zygotes, which can result in mosaicism (multiple alleles), and somatic cell nuclear transfer (SCNT), where the oocyte nucleus is replaced by a nucleus from a somatic cell culture that is transfected with CRISPR/CAS9 [51, 52]. In addition, other site-specific nucleases, such as zinc finger nucleases (ZFNs) and transcription activator-like effector nucleases (TALENs), can also be used. Genetic modifications were also introduced to address PERVs, as the primary cross-species virus of concern. It is only necessary to remove enough of the retrovirus to prevent replication; leaving behind certain membrane proteins may protect the pig from similar retroviral infections. Comparative genomics was integral in finding the retrovirus in the pig genome, using multiple sequence alignments to compare them, designing the gene editing, and assessing the results of gene editing [53].

Several carbohydrate antigens in pigs, such as the Gal antigen, can cause immediate hyperacute xenograft rejection, the quickest and most severe rejection mechanism. However, experiments have identified ways these antigens can be removed [54]. In addition, the introduction of human complement inhibitor genes (CD55, CD46, CD59) has enabled the prevention of complement-mediated xenograph injury [55]. Besides hyperacute xenograft rejection, there are other rejection mechanisms that can occur: coagulation dysregulation, natural killer (NK) cell-mediated cytotoxicity, macrophage-mediated cytotoxicity, and T cell response. Coagulation dysregulation occurs from incompatibilities between pig and human coagulation factors which can be mitigated by introducing human proteins into the pig genome. To reduce NK cytotoxicity, expression of human leucocyte antigens (HLA-E) in pig has been shown to reduce the xenogeneic NK response. These genetic alterations can be performed at the germline-level, resulting in pigs with normal physiology that can successfully reproduce and propagate germline transmission of the edited alleles.

Comparative genomics can help uncover the pathways and molecules responsible for immune rejection, how they have evolved, and conserved and divergent sequences between species [52]. Gene annotations are useful in evaluating differences within and across species, such as in coagulation factors or to further explore immune region, which may in turn lead to future improvements in the annotation of this key genomic region. By comparing sequences, researchers can find novel genes as well as changes between species, such as the protein binding specificity for glycosylation that contributes to immune rejection. Since the xenotransplants will be tested in non-human primates, host genomes can be analyzed to identify factors that contribute to transplant rejection. Latent viruses in the donor genome can also be identified, and multiple genetically altered pig genomes can be compared to assess their clinical outcomes as donors. As transplantation research continues, a key role for comparative genomics will be to understand the mechanisms of longer-term rejection and to continue to push the boundaries for survival for transplant recipients. Creation of the xenotransplant donor transgenic pig is an iterative process; as the most severe immune rejection barriers are edited or augmented, discovering new rejection barriers, and developing strategies to mitigate their effects will be a key challenge.

### Oncology

The genetics of cancer susceptibility is a pivotal health-related research area that has substantially benefited from comparative and evolutionary analysis. Since each cell is potentially vulnerable to mutation, cancer risk is thought to be associated with the number of cells in an organism, leading to a positive relationship between body size and cancer within a species. However, this relationship is not true when considering the differences between species, resulting in a stable intra-species cancer incidence rate [56, 57]. This observation led researchers to identify Afrotherian mammals as an ideal system for investigating cancer-resistance mechanisms as this group contains large-bodied species (e.g., elephants) phylogenetically nested amongst much smaller-bodied species (e.g., elephant shrews).

Using comparative approaches, elephants were found to possess enriched duplication of gene families related to anti-cancer cellular phenotypes, specifically regaining function in a Leukemia-inhibiting pseudogene, and evolving additional copies of tumor suppressor TP53 [57, 58]. A follow-up study found that other Xenarthran mammals, including armadillos and sloths, have convergently evolved similar solutions to mitigating cancer risk, suggesting this group should be studied as models for cancer protection [59]. In general, the application of comparative genomics to cancer genetics has proved fruitful, notably in studies that examine selective pressure across all mammals on cancer-related genes such as BRCA1/2 [60].

Dogs (breeds of *Canis familiaris*) are already used by comparative oncologists as valuable models for the study and treatment of human cancers. Their history of selective breeding makes them particularly suitable for comparative genomics, as it has led to breed-specific genetic diseases that can be used as models for phenotypes not seen in more traditional models, such as mice [61]. Additionally, many dog cancers are strikingly similar to those in humans, allowing for some direct inferences between studies in dogs and outcomes in humans. For example, the genetics of osteosarcoma progression in dogs versus humans are nearly indistinguishable, giving scientists the much-needed opportunity to study a cancer that is relatively rare in humans [62]. As of March 2023, the National Cancer Institute (NCI) Comparative Oncology Program [63] has multiple open trials for cancer treatments in dogs, including one for osteosarcoma. This program and efforts, such as the National Human Genome Research Institute Dog Genome Project [64] and NCI’s Integrated Canine Data Commons [65], are constantly providing new insights into the genetics of canine disease using the principles of evolutionary genomics.

### Toxicology

Environmental toxins that negatively impact free-living organisms, food chains, and ecosystems directly affect human health. Chemical hazard and safety testing inform the development and refinement of regulatory frameworks around levels of pollution that are considered acceptable and do not pose immediate or long-term health risks. Animal testing has been a cornerstone in the assessment of toxicity and other impacts of chemicals released into the environment, with testing on vertebrates, particularly mammals, driving many of the inferences regarding human health. The 3R principles (Reduction, Refinement, and Replacement) of animals used in research, formulated in the late 1950s [66], have not only inspired legislation aimed at reductions in animal use for the development of consumer products, it has spurred the development of New Approach Methods (NAMs) in chemical safety assessment. NAMs include cell-based in vitro models, Next-Generation Sequencing and omics approaches, and computational modeling of Adverse Outcome Pathways (AOPs).

The International Consortium to Advance Cross Species Extrapolation in Regulation (ICACSER) [67] is developing strategies for more cost-effective toxicity testing aimed at reducing the burden on whole-animal testing in favor of cell-based and computational approaches. A major component of these alternatives is species extrapolation: the use of existing knowledge about one species for inferring effects of chemical exposure on another species. A detailed knowledge of AOPs is key to successful species extrapolation. Specifically, this includes Molecular Initiating Events (MIEs), such as those collected in curated literature databases like ECOTOX [68], and the utilization of bioinformatics approaches. The Sequence Alignment to Predict Across Species Susceptibility (SeqAPASS) Tool [69] has been recommended by the Organization for Economic Cooperation and Development (OECD) for the evaluation of protein conservation in cross-species extrapolation. For example, an integrated analysis of cross-species comparability of interactions between estrogen receptor alpha (ERα) and estrogenic chemicals revealed substantial conservation of ligand-binding properties across vertebrate ERαs, while providing little evidence for functional ERαs in most invertebrate species. This discovery suggests limited susceptibility of invertebrates to ERα agonists and is consistent with experimental toxicology data, with the possible exception of ERαs in annelid worms which require more in-depth investigation [70]. High-quality genome annotation, both structurally and functionally, and the tools for comparative analysis of genome organization will inform the characterization of AOPs and provide a level of asserting species similarity that may exceed what can be achieved via pair-wise sequence comparison. Given the availability of genomic sequencing data for increasingly diverse organisms, species extrapolation techniques may broaden the understanding of environmental toxin impacts beyond only a small number of sentinel organisms and may further help to uncover the role of pollutants in the etiology of human disease.

Functional studies of genes and genomes aim at revealing underlying molecular mechanisms and pathways that determine responses to the environment and are inextricably linked to disease, such as health outcomes after exposure to environmental toxins. Comparative genomics plays a fundamental role both in guiding such studies and interpreting their results.

## Addressing challenges in comparative genomics

**Fig. 4:**
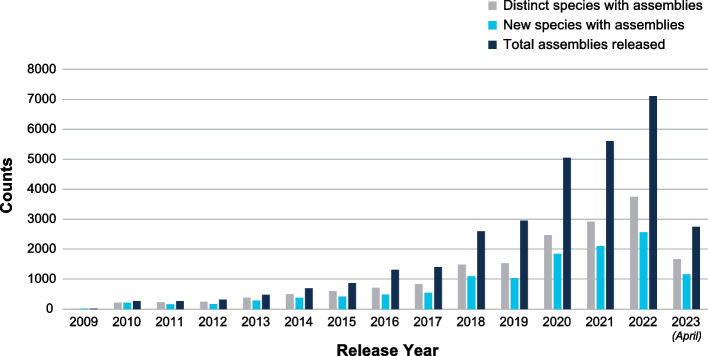
Annual growth in sequenced eukaryotic species and assembled genomes in GenBank

As sequencing methods evolve, the decline in the cost of generating sequence data has outpaced Moore’s law, and there has been a concomitant explosion in the number of eukaryotic genome sequences deposited in publicly-available databases (see Fig. 4) [71, 72]. This growth—in both the numbers of organisms represented and the number of assembled genomes per organism—is essential to improve the resolution of genome comparisons and to fill gaps in the taxonomic tree that would otherwise confound phylogenetic inferences. While resources such as the Generic Model Organism Database (GMOD) project [73] and the Alliance of Genome Resources [74] have provided tools and data resources that harmonize the user experience in accessing and retrieving data for specific organisms, researchers are still faced with multiple diverse interfaces scattered around the web for information on other organisms. This complicates data discovery, integration, and analysis for different organisms, creating research hurdles across all applications towards human health as described above, as well as many others. The growth in data volume is also exerting pressure on the computational hardware and bioinformatics software needed for storage and analysis, upending the traditional model of data retrieval and analysis [75–78]. Multiple sequence alignments are key resources for genomic analysis [79], and the expanding number of genomes creates computational challenges for their generation, driving the development of new methods [72].

Quality variability across genome-associated data also creates challenges for comparative genomics. This unevenness may derive from underlying biological, technical, or human sources. For example, some organisms have genomic features (e.g., high repeat content [80]) or use biological processes (e.g., as trans-splicing [81]) that are not well-supported by common tools for genome assembly or structural annotation. Consequently, corresponding genome-related data is often not available. Differing levels of assembly quality can also drastically impact the ability to annotate genomes and identify gene families of interest in different species. When examining a dataset of draft assemblies compared with their updated versions, up to 40 percent of all gene families had varying numbers and sets of members [82]. In one case, a re-sequencing effort for the genome of the honeybee, *Apis mellifera,* recovered nearly 50 percent more protein-coding genes in the updated genome annotation [83]. Biology and technology together also contribute unevenness in data by creating variability in the quality of assembled genomes, as determined by contamination with foreign sequences [84]; metrics such as completeness (length), contiguity (N50), and base quality (QV) score; and the quality of their corresponding annotations [85–87]. Scientists may also unintentionally introduce unevenness to the analysis landscape for comparative analyses by providing incomplete metadata for the underlying samples that hinders data reuse or results in misinterpretation of results [88]. Most commonly, though, user-sourced unevenness in genome-associated data is a consequence of the organisms chosen for sequencing [89, 90]. These various inconsistencies can have large consequences for phylogeny inferences, identification of syntenies and ortholog definitions [82, 83, 91, 92], and even for practical applications like drug development [93]. Other factors contributing to unevenness are the heterogeneous nature of DNA sequencing technologies, which have different kinds of errors and trade-offs; the software used to process the data; the skill and experience of the molecular biologists who prepare sequencing libraries; and the quality goals of the lab performing the sequencing (primarily how much time, effort, and money they are willing to spend on a sequencing project).

Despite the barriers to scientific advancement, these challenges will likely be met as new technologies are developed to accommodate the sequencing and assembly of complex biological features [94, 95]. For example, large sequencing efforts, such as the Darwin Tree of [96] and Earth Biogenome Project [97], will fill taxonomic-specific data gaps. The growth in the number of sequenced organisms also provides important new data supporting contamination detection and informing relevant software tools. Consequently, the data contamination issue may diminish over time as more accurate detection methods evolve, such as the publicly-available CGR-associated foreign contamination screening (FCS) tool [98]. The NCBI Eukaryotic Genome Annotation Pipeline (EGAP) [99] provides high-quality annotations for a wide range of taxa. As part of CGR, this tool is being made publicly-available to promote high-quality annotation on submitted assemblies. As both the quality of assemblies and their annotations improve, comparative analyses involving increasing numbers of organisms should reveal new biological relationships that inform our understanding of human health. Cloud compatible tools, as well as cloud-based bioinformatics platforms, are emerging as important industry resources for creating workflows for analysis of large volumes of genomic data, such as those involved in comparative genomics [100–104]. The need for continued tool development and new approaches to bioinformatics analyses align with CGR.

CGR will play a crucial role in bringing together research communities through an organism-agnostic approach and provide easy access to sequencing projects from different consortia, thereby facilitating cross-species analyses. For example, a researcher working on a phenotype, such as aging or cancer, will be able to download sequences more easily because they will have identified a relevant and broad array of organisms that display phenotypes of interest. Although creating and maintaining organism-specific resources for every newly-sequenced organism is expensive and untenable, CGR can offset that burden by providing organism-agnostic resources to meet the needs of communities for which it is not cost-effective to create and maintain organism-specific resources. By engaging with existing genomics resources, CGR can raise awareness of their assets in additional organismal communities. NCBI Datasets, a new resource supporting CGR, provides web and programmatic interfaces to aid in the discovery of genomic data and metadata stored in multiple NCBI databases, and delivers these data in a coherent package that can contain information for large numbers of genomes and species, including all those described in the human health use cases above [105, 106]. These data are made available continuously through NCBI Datasets as they are released into the public domain from the GenBank and RefSeq, their source databases, with more than 32,000 eukaryotic assembled genomes already included.

CGR ensures greater quality and standardization of data, which increases the confidence in comparative genomics findings. Existing and forthcoming tools including EGAP and FCS, facilitate accurate and far-reaching species extrapolation and functional studies. Access to these tools, combined with the organism-agnostic repositories of NCBI Datasets, may also help alleviate gaps in sequencing diversity by easing the burden for smaller, organism-specific sequencing groups with fewer resources. The extension of high-quality genome annotation from model organisms to a wider array of vertebrate and non-vertebrate species supports successful cross-species extrapolation. Tools such as ClusteredNR, a new BLAST database, and the Comparative Genomics Viewer (CGV) [107] aid with cross-species comparisons through reliable and consistent orthology assignments. These tools also facilitate sequence visualization for cross-species comparisons that can shed light on evolutionary trajectories including retroviral genes in pig, human, and other species (i.e., Reverse Transcriptase [RT] genes) in xenotransplantation research.

NCBI is developing multiple use cases to illustrate how the community can leverage CGR to advance their research. One example involves similar TP53 research https://ncbiinsights.ncbi.nlm.nih.gov/2023/06/14/canine-human-oncology-cgr/ as referenced above. In dogs, TP53 has been linked to osteosarcoma and histiocytic sarcoma. In addition to using longstanding elements of NCBI that support comparative genomics, such as the Genome Data Viewer (GDV), COBALT multiple sequence alignment, and the iCn3D structure viewer, researchers can leverage the NCBI toolkit within CGR to explore the dog TP53 gene and its variants through NCBI Gene and compare syntenic regions between dogs and humans in CGV.

Another use case that will be shared though CGR outreach explores how CGR and other NCBI resources can be applied to streamline the gathering and comparison the sequence data necessary to assess whether particular non-mammal animals (for example, songbirds) are susceptible to and could be a vector for SARS-CoV-2. This research can be accomplished in part with ClusteredNR, which makes BLAST results more concise and more representative of organismal diversity, informative in identifying genes of interest, such as ACE2, in potential animal vectors. To further enhance this work, researchers can use the new NCBI Datasets Genome hub to easily assess genomic data availability for taxa of interest (e.g., songbirds), and filter these results by metadata such as availability of annotation, in order explore the potential for these organisms to act as reservoirs. The new NCBI Datasets command line interface and API can also be used to accomplish these tasks in a research workflow, such as one to find candidate SARS-CoV-2 vector species. Additionally, as more cross species alignments become available in CGV, synteny can be assessed to explore for genomic structural variation that may impact their susceptibility. Precomputed orthologs for ACE2, the SARS-CoV-2 receptor can be searched, viewed, and downloaded by taxonomic group, such as songbirds, and their domain organization explored via CD-Search, which may provide researchers with further insights into this question. Tasks such as these are common to comparative genomics. As NCBI continues to support the improvement of its comparative genomics resources and expand connections to and from community-provided resources, the impact of these technologically advanced genomics tools will continue to grow and be reflected in the published literature.

## Conclusion

Human diseases and other challenges to human health can be viewed as products of an interplay between pathways and systems originating deep in evolutionary time, as well as more recent lineage-specific changes [108]. Fully understanding contributors to human health (particularly with regards to genetics), requires the application of evolutionary principles and the study of organisms both closely and distantly related to humans. On average, broad surveys of the genome reveal that genes implicated in human disease are more ancient than the rest of the human genome, and that genes have varying evolutionary ages, suggesting that different organisms may be suitable for the study of different genes [109]. Additionally, organisms throughout the tree of life have evolved solutions to issues of biomedical relevance and display a huge variation in relevant phenotypes such as lifespan and aging [110]. Applying evolutionary principles to study this diversity may, ultimately, lead to the development of new model systems for key genetic pathways and phenotypes.

Comparative genomics offers unique and critical insights into many aspects of human health; therefore, it is vital to find solutions that overcome the challenges in performing this research. CGR aims to maximize the impact of eukaryotic research organisms and their genomic data resources to biomedical research. CGR facilitates reliable comparative genomics analyses for all eukaryotic organisms through community collaboration and a NCBI genomics toolkit. The toolkit provides high-quality data, tools, and interfaces for connecting community-provided resources with NCBI. The organism-agnostic tools and resources have vast implications both as described above and beyond. These tools will be crucial in identifying emerging model organisms to address new applications to human health, cataloging and investigating evolution and biodiversity, and accelerating scientific advancement. A catalog of genomes across the tree of life will also be integral, when combined with advances in artificial intelligence (AI) in the emerging science of synthetic biology— offering the capability for designing innovative proteins and drugs to meet our most pressing human health needs.

## Data Availability

Not applicable.

## Abbreviations

3R: Reduction, Refinement, and Replacement
ACE2: Angiotensin Converting Enzyme 2
ADAM: A Database Of Anti-Microbial Peptides
ADME: Absorption, Distribution, Metabolism, Excretion
AGR: Alliance Of Genome Resources
AMD: Antimicrobial Peptide Database
AMP: Antimicrobial Peptide
AOP: Adverse Outcome Pathways
BAC: Bacterial Artificial Chromosome
BLAST: Basic Local Alignment Search Tool
BNTL: Butyrophilin-like genes
BRCA1: Breast Cancer Gene 1
BRCA2: Breast Cancer Gene 2
CAMPR3: Collection of Antimicrobial Peptides Release 4
CD45: Complement Decay Accelerating Factor 45
CD56: Complement Decay Accelerating Factor 56
CD59: Complement Decay Accelerating Factor 59
CGR: Comparative Genomics Resource
CGV: Comparative Genomics Viewer
ClusteredNR: Clustered Non-Redundant Database
CRISPR/CAS9: Clustered Regularly Interspaced Short Palindromic Repeats/ CRISPR-associated protein 9
COVID-19: Coronavirus Disease of 2019
DBAASP: Database of Antimicrobial Activity and Structure of Peptides version 3
DRAMP 3.0: Data Repository of Anti-Microbial Peptides version 3
ECOTOX: Toxicology Knowledgebase from Environmental Protection Agency
EGAP: Eukaryotic Annotation Pipeline
ERα: Estrogen Receptor Alpha
EID: Emerging Infectious Disease
FCS: Foreign Contamination Screening Tool
GAL: Galactose-α(1,3)-Galactose
GMOD: Generic Model Organism Database
H1N1: Influenza Virus with Hemagglutinin 1 and Neuraminidase 1
HLA: Human Leucocyte Antigen Complex
HLA-E: Human Leucocyte Antigen E
HIV: Human Immunodeficiency Virus
ICACSER: International Consortium to Advance Cross Species Extrapolation in Regulation
LAMP2: Linking Anti-Microbial Peptides 2 (database)
MHC: Major Histocompatibility Complex
MIE: Molecular Initiating Event
MOA: Mechanism Of Action
NAM: New Approach Methods
N50: Contiguity statistic for assembled DNA sequence
NCBI: National Center for Biotechnology Information
NCI: National Cancer Institute
NIH: National Institutes of Health
NK: Natural Killer cells
NLM: National Library Of Medicine
OECD: Organization For Economic Cooperation and Development
PERV: Porcine Endogenous Retrovirus
SARS-CoV-2: Severe Acute Respiratory Syndrome Coronavirus 2
SCNT: Somatic Cell Nuclear Transfer
SLA: Swine Leucocyte Antigen Complex
TALEN: Transcription Activator-Like Effector Nuclease
TP53: Tumor Protein 53
SeqAPASS: Sequence Alignment to Predict Across Species Susceptibility
QV: Base quality score for assembled DNA sequence
WHO: World Health Organization
YPS7: An aspartyl protease
